# Distinct Role of IL-27 in Immature and LPS-Induced Mature Dendritic Cell-Mediated Development of CD4^+^ CD127^+^3G11^+^ Regulatory T Cell Subset

**DOI:** 10.3389/fimmu.2018.02562

**Published:** 2018-11-13

**Authors:** Fang Zhou, Guang-Xian Zhang, Abdolmohamad Rostami

**Affiliations:** Department of Neurology, Thomas Jefferson University, Philadelphia, PA, United States

**Keywords:** dendritic cell, immune tolerance, immunotherapy, regulatory T cell, IL-27

## Abstract

Interleukin-27 (IL-27) plays an important role in regulation of anti-inflammatory responses and autoimmunity; however, the molecular mechanisms of IL-27 in modulation of immune tolerance and autoimmunity have not been fully elucidated. Dendritic cells (DCs) play a central role in regulating immune responses mediated by innate and adaptive immune systems, but regulatory mechanisms of DCs in CD4^+^ T cell-mediated immune responses have not yet been elucidated. Here we show that IL-27 treated mature DCs induced by LPS inhibit immune tolerance mediated by LPS-stimulated DCs. IL-27 treatment facilitates development of the CD4^+^ CD127^+^3G11^+^ regulatory T cell subset *in vitro* and *in vivo*. By contrast, IL-27 treated immature DCs fail to modulate development of the CD4^+^CD127^+^3G11^+^ regulatory T cell sub-population *in vitro* and *in vivo*. Our results suggest that IL-27 may break immune tolerance induced by LPS-stimulated mature DCs through modulating development of a specific CD4^+^ regulatory T cell subset mediated by 3G11 and CD127. Our data reveal a new cellular regulatory mechanism of IL-27 that targets DC-mediated immune responses in autoimmune diseases such as multiple sclerosis (MS) and experimental autoimmune encephalomyelitis (EAE).

## Introduction

Interleukin-27 (IL-27) is an important cytokine that plays a critical role in regulation of immune responses *in vivo* (1). Previous research has shown that IL-27 is an anti-inflammatory cytokine. For example, it blocks Th17-mediated immune responses (2). However, recent data also indicate that IL-27 plays the role of an inflammatory cytokine that facilitates T cell-mediated immune responses (3–5). These contradictory results suggest the complex regulatory mechanisms of IL-27 *in vivo*(1).

CD127 is a subunit of interleukin-7 (IL-7) receptor. It is composed of 459 amino acids and is expressed in mature T cells, monocytes and macrophages. In particular, CD127 is a biomarker of regulatory T cells (T_regs_) (6, 7). Several sub-populations of T_regs_ have been defined according to the importance of CD127 expression on CD4^+^ T cells (8).

3G11 is a membrane antigen expressed on murine CD4^+^ T cells. 3G11 is a ganglioside with mobility between GD1a and GD1b complexes in the human brain. 3G11 antigen is identified as the disialoganglioside IV3(NeuAc)2-GgOse4Cer. The immune function of 3G11 on CD4^+^ T cells has not been fully elucidated. Recent research demonstrated that 3G11 may be a biomarker of T_reg_ subsets (8–13).

Dendritic cells (DCs) are important immune regulatory cells that play a central role in development of T cells such as CD4^+^ T helper cells. DCs modulate development and differentiation of T cells through production of multiple cytokines such as IL-27 (5). It is not clear whether IL-27 can affect DC-mediated CD4^+^ T cell immune responses. The effect of IL-27 on development of CD4^+^ regulatory T cells was examined in this project. Our data show that IL-27 modulates development of mature DC-mediated differentiation of T_reg_ subsets.

Regulatory T cells are important immune cells *in vivo* and they play a central role in induction of immune tolerance and anti-inflammatory responses (14, 15). Multiple subsets of T_regs_ have been reported. For example, our previous data show that there are two subpopulations of T_regs_ including CD4^+^CD25^+^FoxP3^+^GITR^+^CD127^+^3G11^+^ and CD4^+^CD25^+^FoxP3^+^GITR^+^CD127^+^3G11^−^ T_reg_ subsets *in vivo*. Although the immune function of these two new subsets of T_regs_ is unclear, the number of T_reg_ sub-populations mediated by 3G11 and CD127 is different in mice with experimental autoimmune encephalomyelitis (EAE) development and those with immune tolerance. However, the regulatory mechanisms of CD4^+^CD127^+^3G11^+^ T_regs_ are still unclear (8). Our project is focused on whether or not IL-27 plays an important role in the development of CD4^+^CD127^+^3G11^+^ T_regs_ mediated by immature or mature DCs induced by LPS. Our results will show that IL-27 modulates development of CD4^+^CD127^+^3G11^+^ T_regs_ mediated by mature DCs, and they may help to reveal a new mechanism of IL-27 in mature DC-mediated immune responses.

## Materials and methods

### Mice

Wild type C57 BL/6J female mice (8–12 weeks) were purchased from The Jackson Laboratory (Bar Harbor, ME, USA). All mice were bred in Thomas Jefferson Animal Care facilities and all experimental procedures were approved by the Institutional Animal Care and Use Committee of Thomas Jefferson University.

### Immunogen and peptide

Mouse MOG_35−55_ peptide (MEVGWYRSPFSRVVHLYRNGK), an ingredient of myelin oligodendrocyte glycoprotein (MOG), was obtained from Invitrogen (Invitrogen, Carlsbad, CA, USA).

### Isolation of bone marrow

As described previously, femurs and tibiae were isolated from muscle tissue of mice. The intact bones were then sterilized with 70% ethanol for 5 min and washed with phosphate-buffered saline (PBS). Bone ends were cut and the bone marrow was flushed with PBS. Cellular clusters within the bone marrow suspension were disintegrated and washed with PBS (8, 16–20).

### Bone marrow-derived DC culture

As described previously, leucocytes from bone marrow were fed in bacteriological 100 mm Petri dishes (Falcon, Becton Dickinson, Heidelberg, Germany) at 2 × 10^6^ cells per dish. Cells were cultured in RPMI1640 complete medium (Gibco-BRL, Eggenstein, Germany) including penicillin (100 U/ml, Sigma, St. Louis, MO, USA), streptomycin (100 U/ml, Sigma), L-glutamine (2 mM, Sigma), 2-mercaptoethanol (2-ME, 50 μM, Sigma), 10% heated, inactivated and filtered (0.22 μm, Milipore, Inc., Bedford, MA, USA) Fetal Calf Serum (FCS, Sigma) and granulocyte-macrophage colony-stimulating factor (GM-CSF, PeproTech, Rocky Hill, NJ, USA) at 20 ng/ml at day 0 (10 ml medium per dish) (8, 16–20).

At day 3, 10 ml fresh medium with GM-CSF (20 ng/ml) was added to each dish and, at day 6, half of the medium (about 10 ml supernatant) was collected and centrifuged at 300 g for 5 min. Subsequently, cells were re-suspended in 10 ml fresh medium with GM-CSF (20 ng/ml) and were then re-fed in the original dish. Only non-adherent cells (DCs) were harvested and seeded in a fresh dish; 10 ml fresh medium including GM-CSF (20 ng/ml) was added at day 8 (8, 16–20).

Cells were also treated with lipopolysaccharide (LPS, Sigma) for 24 h at 1 μg/ml. LPS was isolated from *K. Pneumoniae*. DCs or LPS-treated DCs were pulsed with 0.1 μM MOG peptide for 30 min and then washed twice with PBS at 300 g × 5 min before i.v. transfer to EAE mice. DCs were treated with IL-27 at 100 ng/ml for 72 h before conducting flow cytometry assay or i.v transfer experiments. Fresh non-adherent DCs were then collected and washed with PBS at 300 g for 5 min and characterized by flow cytometry or i.v. transferred to EAE mice. More than 90% of cells expressed DC marker CD11c (8, 16–20).

### Flow cytometry

MOG-primed T lymphocytes were isolated from EAE mice and incubated with anti-mouse CD4 (Pacific blue), CD25 (APC), CD127 (PerCP-Cy5.5), 3G11 (PE-Cy7) and GITR (APC-Cy7) antibodies (Biolegend). Cells were washed twice with 5% FCS in PBS at 300 g for 5 min, fixed with 5% formalin in PBS at 4°C for 2 h and then permeated for intracellular staining (8, 16–20).

For intracellular staining, spleen cells were stimulated by leukocyte activator (BD) for 6 h. Splenocytes were then washed twice with 5% FCS in PBS at 300 g for 5 min and fixed with 5% formalin (Sigma) in PBS at 4°C for 2 h. After cells were washed with permeabilization buffer (Biolegend) twice at 300 g × 10 min, anti-mouse FoxP_3_ (PE) antibody (Biolegend) was incubated with cells at 4°C for 24 h. Cells were then washed with permeabilization buffer twice at 300 g for 5 min, re-suspended in 0.5 ml cell staining buffer (Biolegend), and tested in a FACSAria (BD Biosciences, San Jose, CA, USA). Data were analyzed using FlowJo software (Treestar, Ashland, OR, USA) (8, 16–20).

### Generation of effector T cells *in vitro*

C57 BL/6J mice were immunized with MOG_35−55_ peptide (Invitrogen) 200 μg, QuilA (Sigma) 20 μg, and keyhole limpet hemocyanin (KLH, Sigma) 20 μg per mouse at day 0. Spleen cells were then isolated at day 10 after immunization. CD4^+^ T lymphocytes were purified with mouse CD4^+^ T cell subset column kit (R&D Systems). CD4^+^ T cells (1 × 10^6^ cells/per well) were co-cultured with DCs at 10:1 (T cells: DCs) and pulsed with MOG _35−55_ peptide at 0.1 μM in complete medium with mouse IL-2 at 1 ng/ml for 3 days. Cells were harvested and MOG-primed CD4^+^ T cells were gated and analyzed by flow cytometry (8, 16–20).

### EAE induction and treatment

C57BL/6J mice (female, 8–12 week) were immunized with MOG_35−55_ peptide/complete Freund's adjuvant (CFA, Sigma) at 200 μg/200 μl/per mouse (subcutaneous injection, s.c.). Pertussis toxin (PT, Sigma) was simultaneously injected at 200 ng/per mouse (intraperitoneal injection) and the second PT injection was conducted after 48 h. EAE was assessed following standard clinical scores: 0.5: paralysis of half the tail, 1: paralysis of whole tail, 2: paralysis of tail and one leg, 3: paralysis of tail and two legs, 4: moribund, 5: death (8, 16–20).

Mice were divided into five groups. DCs were washed with PBS twice and were immediately injected via tail vein (3 × 10^5^ cells/per mouse/per time) on days 11, 14, and 17 post-immunization (p.i): (1) injected with PBS only (EAE control); (2) injected with DCs pulsed with MOG peptide; (3) injected with IL-27-treated DCs pulsed with MOG peptide; (4) injected with LPS-DCs pulsed with MOG peptide; (5) injected with LPS and IL-27-treated DCs pulsed with MOG peptide (8, 16–20).

At day 25 p.i., splenocytes were isolated and stimulated with MOG peptide (0.1 μM) and mouse IL-2 (1 ng/ml) for 3 days. Cells were then harvested for flow cytometry assay (8, 16–20).

### Statistical analysis

Experimental data were analyzed using Prism software (GraphPad, La Jolla, CA, USA). A *two-way ANOVA* test was performed for analysis of clinical score of EAE; *t* tests were conducted for analysis of flow cytometry data. Error bars represent the mean and standard deviation (SD) or standard error of arithmetic mean (SEM). Results are considered to show a significant difference if the P value is less than 0.05 (8, 16–20).

## Results

### IL-27-treated immature DCs do not affect expression of T_reg_-associated molecules on CD4^+^ T cells

**Figure 1:**
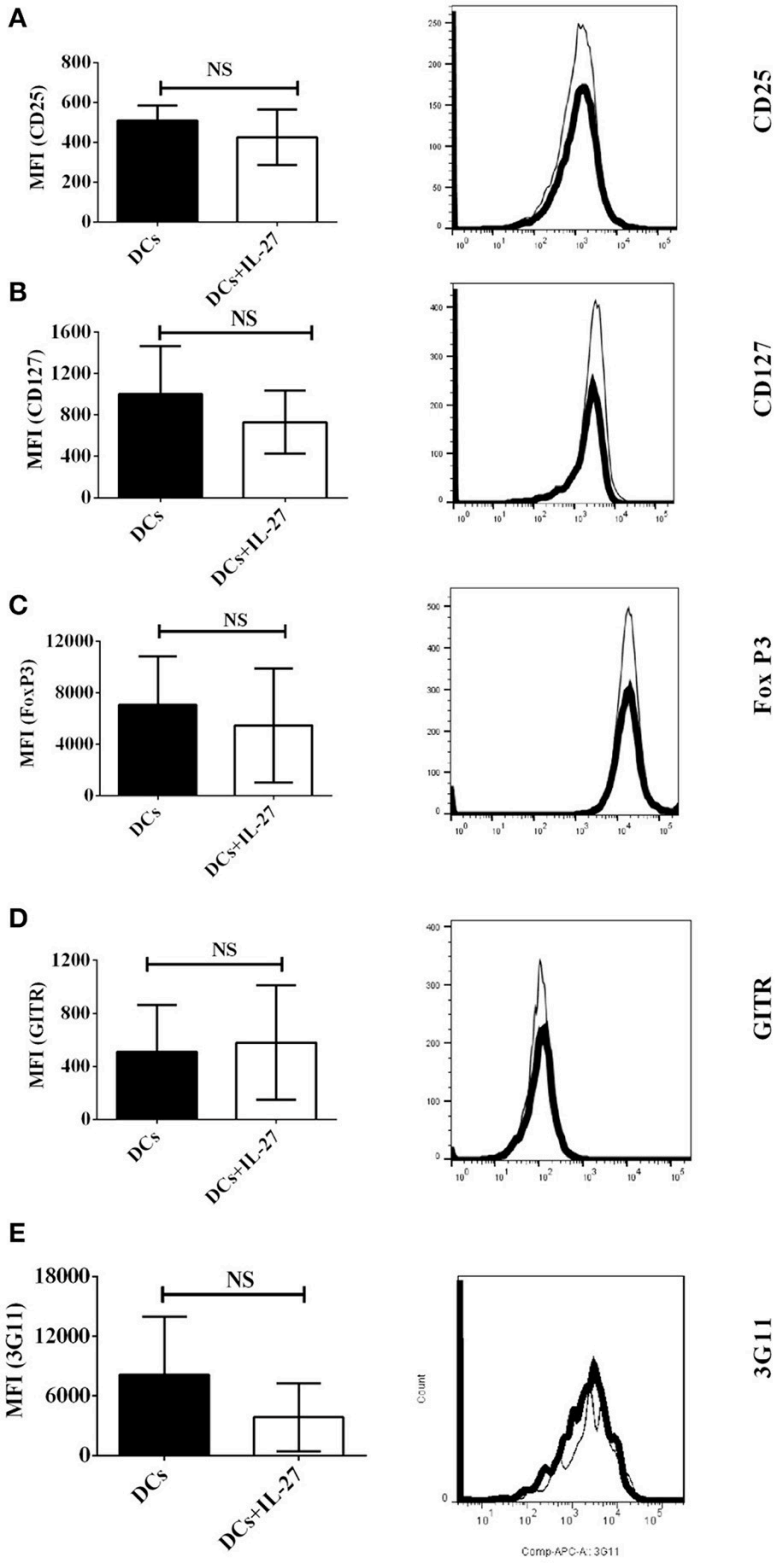
Expression of T_reg_-associated molecules on MOG-primed CD4^+^ T cells co-culture with immature DCs (Thin line) or IL-27-treated immature DCs (Thick line) pulsed with MOG peptide *in vitro*. C57 BL/6J mice were immunized with MOG (200 μg)/Quil A (20 μg) / KLH (20 μg)/per mouse at day 0. Splenocytes were harvested at day 10. CD4^+^ T cells were then isolated using mouse CD4^+^ T cell subset column kit (R and D Systems). CD4^+^ T Lymphocytes were re-stimulated with MOG peptide (0.1 μM) and IL-2 (1 ng/ml) for 72 h. Cells were then stained by anti-mouse CD25 **(A)**, CD127 **(B)**, FoxP3 **(C)**, GITR **(D)**, and 3G11 **(E)** antibodies. Protein expression of T_reg_-associated molecules on CD4^+^ T cells is shown. Error bars indicated in this figure represent mean and SD of triplicate determinations of mean fluorescence intensity (MFI) of T_reg_-associated molecule expression on CD4^+^ T cells (*n* = 3, *t* test, P_A_ = 0.4198; P_B_ = 0.4450; P_C_ = 0.6047; P_D_ = 0.8372; P_E_ = 0.2523; NS, no significant difference).

Since CD25, CD127, FoxP3, GITR, and 3G11 are T_reg_-associated molecules and expressed on CD4^+^ T cells, we supposed that IL-27-treated DCs may affect expression of CD25, CD127, FoxP3, GITR, and 3G11 on CD4^+^ T cells and then regulate development of T_regs_ via modulating expression of T_reg_-associated molecules. To test whether IL-27-treated immature bone marrow-derived DCs can affect protein expression of T_reg_-associated molecules on MOG-primed CD4^+^ T cells, DCs (Thin line) or IL-27-treated DCs (Thick line) were pulsed with MOG peptide and co-cultured with MOG-primed CD4^+^ T cells. The expression of CD25, CD127, FoxP3, GITR, and 3G11 on CD4^+^ T cells co-cultured with MOG-loaded DC or MOG-pulsed DC (MOG-DCs) treated with IL-27 is shown (Figures 1A–E). The experimental data indicate that there is no significant difference in expression of T_reg_-associated molecules on CD4^+^ T cells incubated with MOG-DCs or MOG-DCs-treated with IL-27.

### Nor do IL-27-treated mature DCs induced by LPS affect expression of T_reg_-associated molecules on MOG-primed CD4^+^ T cells

**Figure 2:**
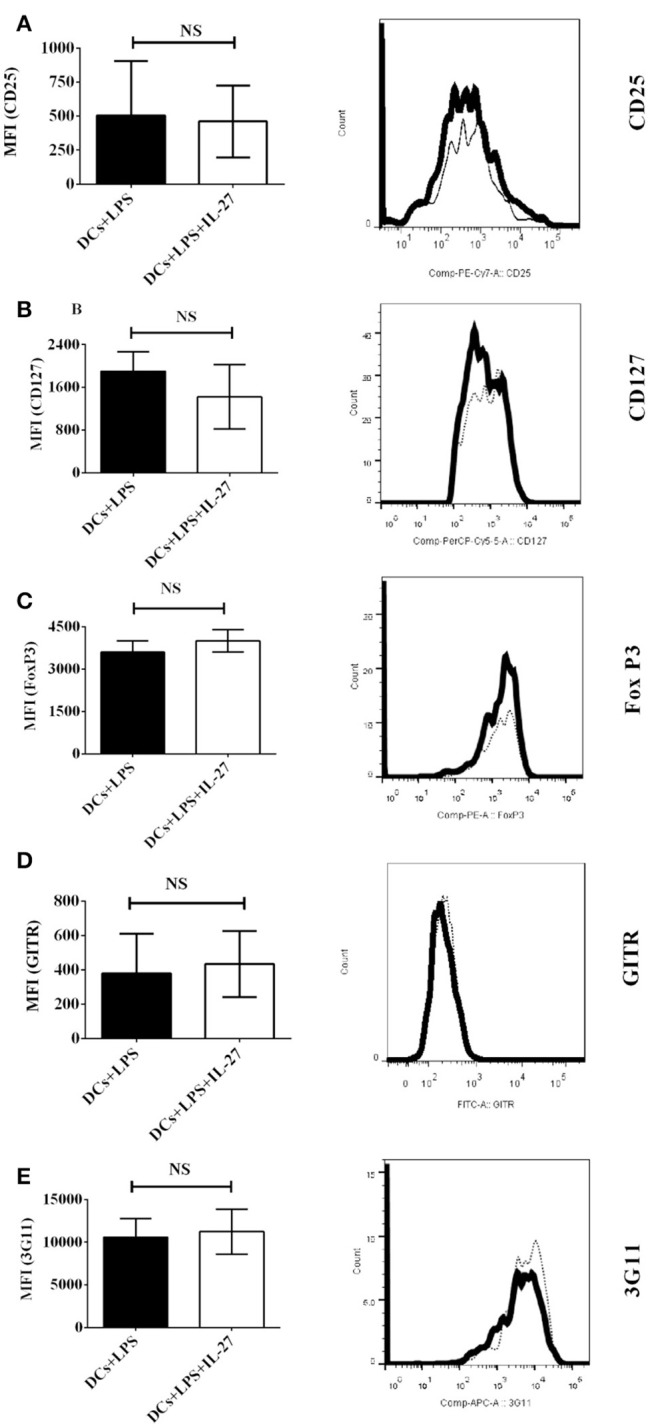
Protein expression of T_reg_-associated molecules on MOG-primed CD4^+^ T cells incubated with LPS-induced mature DCs or IL-27-treated mature DCs pulsed with MOG peptide *in vitro*. Bone marrow-derived dendritic cells were stimulated with LPS (1 μg/ml) for 24 hrs. LPS-stimulated DCs were also simultaneously incubated with IL-27 (20 ng/ml) (Thick line) for 72 hrs or had no IL-27 treatment (Dot line). DCs were then co-cultured with MOG-primed CD4^+^ T cells as shown in Figure 1. Protein expression of CD25 **(A)**, CD127 **(B)**, FoxP3 **(C)**, GITR **(D)**, and 3G11 **(E)** on CD4^+^ T cells is shown. Error bars indicated in this figure represent mean and SD of MFI of T_reg_-associated molecules expressing on CD4^+^ T cells in three independent experiments (*n* = 3, *t* test, P_A_ = 0.8809; P_B_ = 0.3012; P_C_ = 0.2879; P_D_ = 0.7744; P_E_ = 0.7549; NS, no significant difference).

Although IL-27-treated immature DCs do not affect protein expression of T_reg_-associated molecules on CD4^+^ T cells (Figure 1), it is unclear whether or not mature DCs induced by LPS can do that. To detect whether or not IL-27 treatment can modulate mature DC-mediated expression of T_reg_-associated molecules on CD4^+^ T cells, MOG-pulsed mature bone marrow-derived DCs induced by LPS were treated with IL-27 (Thick line) or without IL-27 treatment (Dot line) and co-cultured with MOG-primed CD4^+^ T cells. The expression of CD25, CD127, FoxP3, GITR, and 3G11 on CD4^+^ T cells is demonstrated (Figures 2A–E). Our data indicate that expression of T_reg_-associated molecules on CD4^+^ T cells co-cultured with IL-27-treated mature DCs is similar to that on CD4^+^ T cells co-cultured with mature DCs without IL-27 treatment. It can be concluded that IL-27 treatment does not modulate either immature or mature DC-mediated expression of CD25, CD127, FoxP3, GITR, and 3G11 on MOG-primed CD4^+^ T cells.

### IL-27 treatment facilitates development of CD4^+^CD127^+^3G11^+^ T_regs_ mediated by LPS-induced mature DCs

**Figure 3:**
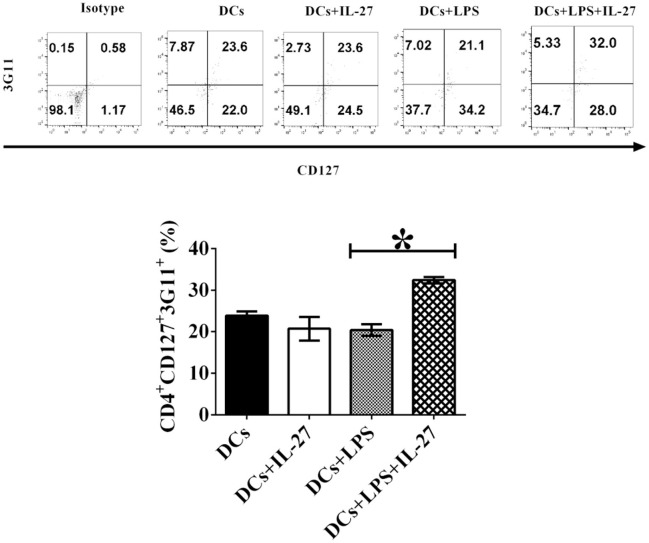
IL-27 treatment facilitates LPS-induced mature DCs-mediated development of CD4^+^CD127^+^3G11^+^ T_reg_ subset *in vitro*. Bone marrow-derived DCs shown in Figures 1, 2 were treated with IL-27 (20 ng/ml, 72 h) or/and LPS (1 μg/ml, 24 h) or without LPS or IL-27 incubation. DCs were then co-cultured with MOG-primed CD4^+^ T cells indicated in Figures 1, 2 for 72 h. CD4^+^CD25^+^FoxP3^+^GITR^+^ cells were gated. The frequency of CD127^+^3G11^+^ T_regs_ is shown. Isotype control is CD4^+^ T cells incubated with isotype control antibodies. Error bars shown in this figure represent mean and SD of frequency of CD4^+^CD127^+^3G11^+^ T_regs_ in three independent experiments (*n* = 3, *t* test, P _(DC, DC+IL−27)_ = 0.1357; P_(DC+LPS, DC+LPS+IL−27)_ = 0.0002).

Although immature and mature DCs treated with IL-27 do not affect expression of T_reg_-associated molecules on CD4^+^ T cells (Figures 1, 2), we assumed that IL-27-treated immature or mature DCs may still modulate development of T_reg_ sub-populations. To test whether IL-27 can affect immature and mature DC-mediated development of CD4^+^ T_reg_ subsets, immature, and mature DCs were incubated with or without IL-27 treatment. Immature and mature DCs were then pulsed with MOG peptide and co-cultured with MOG-primed CD4^+^ T cells. Phenotypes of CD4^+^ T_regs_-mediated by CD127 and 3G11 are shown (Figure 3). The experimental results indicate that immature DCs treated with IL-27 cannot modulate development of CD4^+^CD127^+^3G11^+^ T_reg_ subset; however, LPS-induced mature DCs treated with IL-27 can enhance development of the CD4^+^CD127^+^3G11^+^ T_reg_ sub-population (Figure 3). This suggests that LPS may modulate mature DC-mediated development of CD4^+^ T_reg_ subsets *in vitro*.

### IL-27-treated mature DCs block immune tolerance induced by LPS-stimulated DCs *in vivo*

**Figure 4:**
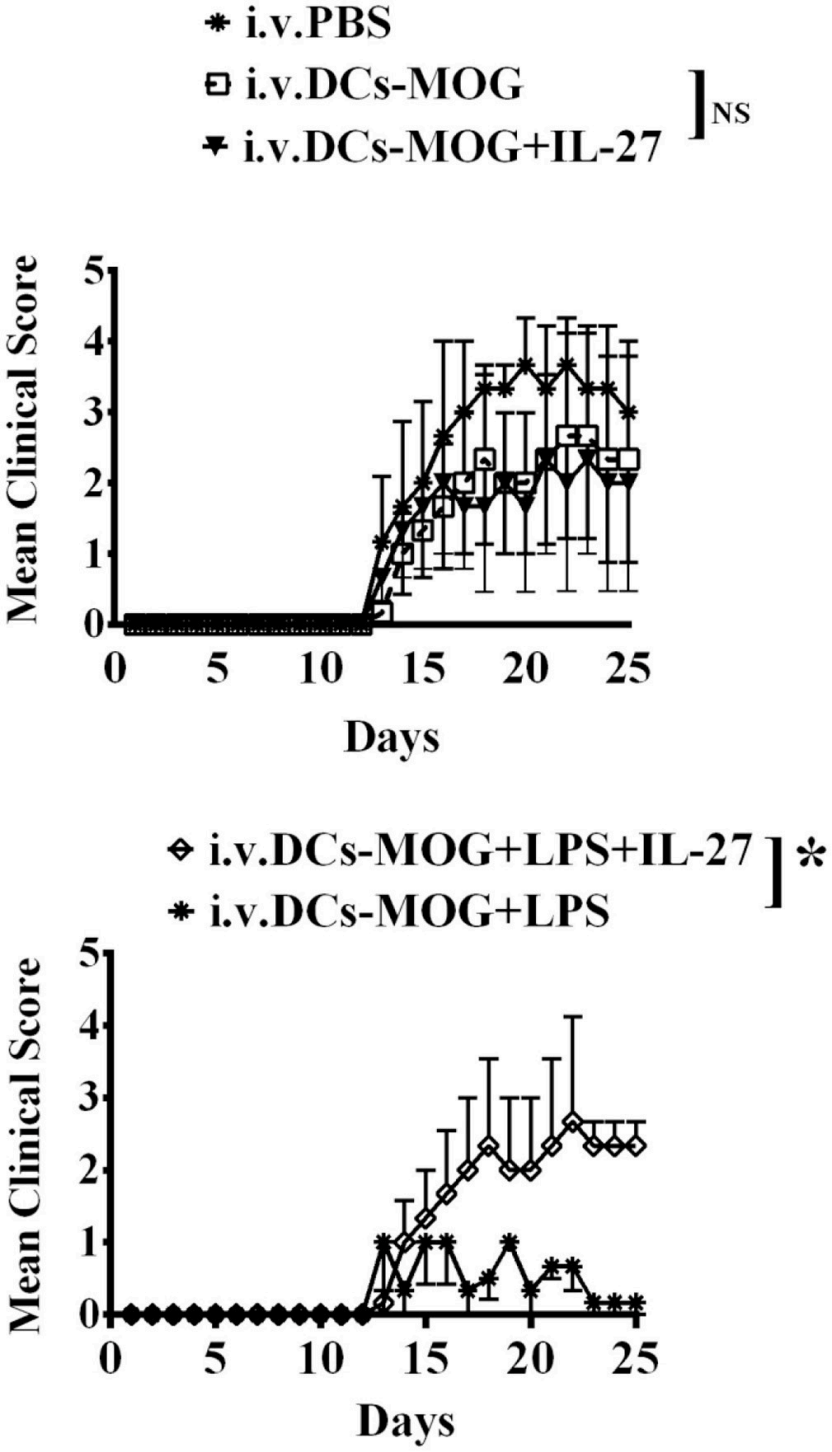
i.v. transfer of IL-27-treated mature DCs induced by LPS inhibits immune tolerance mediated by LPS-stimulated DCs *in vivo*. C57 BL/6J mice were immunized with MOG peptide (200 μg/per mouse) and CFA. Immature dendritic cells (DCs) were incubated with IL-27 (DCs+IL-27) or LPS (DCs+LPS) or both IL-27 and LPS (DCs+LPS+IL-27). Mice in control group were i.v. transferred with PBS. EAE was then induced and shown by clinical score. Error bars in this figure represent mean and SEM of triplicate determinations of EAE clinical score in one experiment (*n* = 3, *two-way ANOVA* test, P _(DC, DC+IL−27)_ = 0.7960; P_(DC+LPS, DC+LPS+IL−27)_ = 0.0001; NS, no significant difference).

We have investigated the effect of immature and mature DCs treated with IL-27 on development of T_regs_ *in vitro* (Figures 1–3). It is necessary for establishment of *in vivo* model to detect whether or not immature and mature DCs treated with IL-27 can regulate development of T_regs_. To test whether or not IL-27 treated immature and mature DCs can affect MOG-primed CD4^+^ T cell-induced autoimmunity *in vivo*, immature and LPS-induced mature DCs were pulsed with MOG peptide and incubated with or without IL-27 treatment. Immature and mature DCs were then i.v transferred into C57BL/6J mice immunized with MOG peptide to induce EAE. Our data indicate that IL-27 treatment blocks immune tolerance mediated by LPS-induced mature DCs. By contrast, IL-27 treatment does not affect the development of EAE in mice that are i. v. transferred with immature DCs (Figure 4). The experimental results suggest that IL-27 may affect mature DC-mediated immune responses but that it does not affect immature DC-mediated immune responses.

### IL-27 treated immature DCs do not affect expression of T_reg_-associated molecules on CD4^+^ T cells *ex vivo*

**Figure 5:**
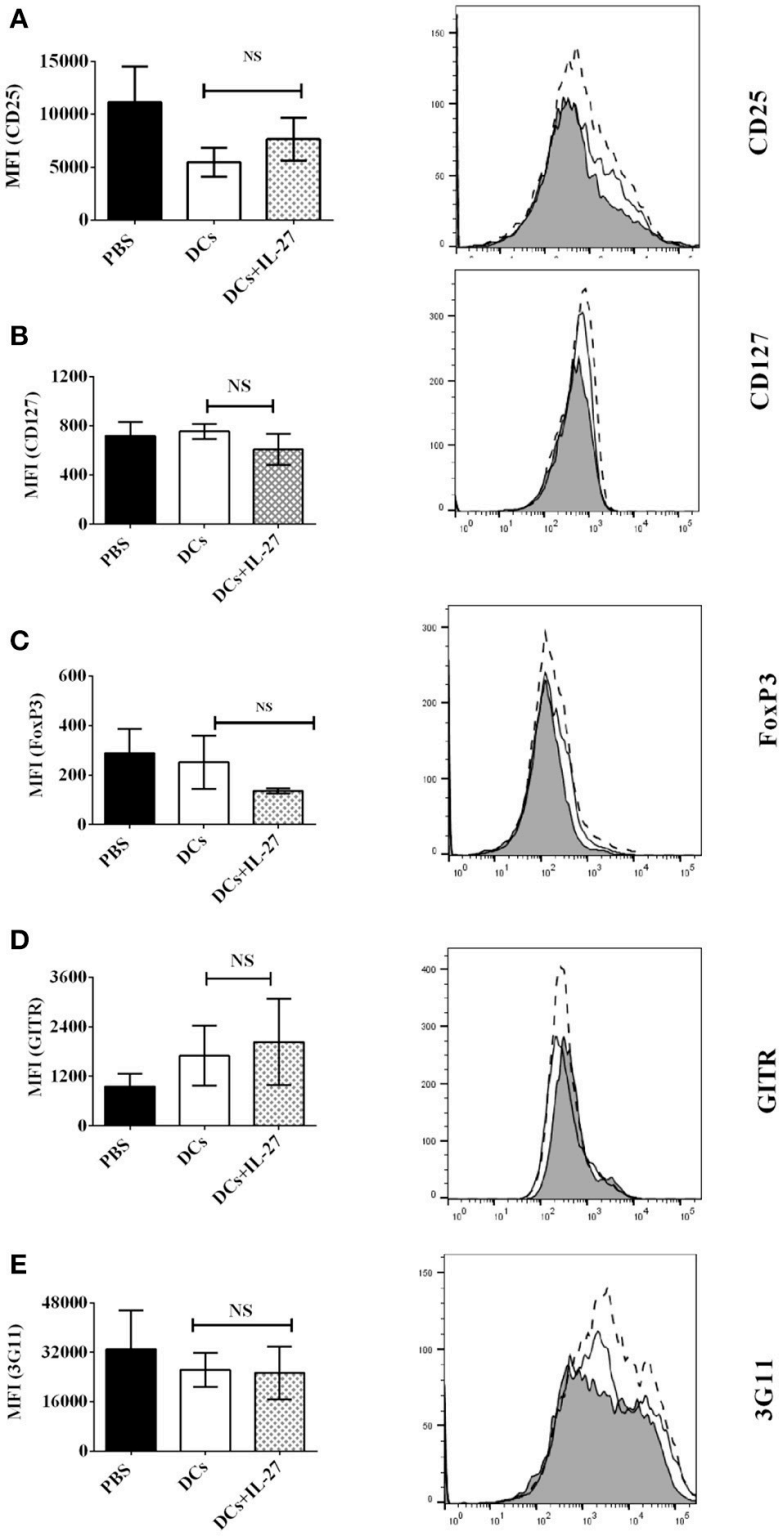
IL-27-treated immature DCs do not affect expression of T_reg_-associated molecules on CD4^+^ T cells *ex vivo*. Spleen cells were isolated from mice treated with PBS (Shade) or DCs (Dot line) or DCs+IL-27 (Thin line) indicated in Figure 4. T lymphocytes were re-stimulated by MOG peptide (0.1 μM) and IL-2 (1ng/ml) for 72 h. The expression of CD25 **(A)**, CD127 **(B)**, FoxP3 **(C)**, GITR **(D)**, and 3G11 **(E)** on CD4^+^ T cells is shown. Error bars demonstrated in this figure represent mean and SD of MFI of T_reg_-associated molecules present on CD4^+^ T cells in three independent experiments (*n* = 3, *t* test, P_A_ = 0.1944; P_B_ = 0.1476; P_C_ = 0.2879; P_D_ = 0.7744; P_E_ = 0.7549; NS, no significant difference).

Our data of *in vitro* assay have shown that IL-27-treated immature DCs do not affect expression of T_reg_-associated molecules on CD4^+^ T cells (Figure 1), however, it is still unknown whether or not IL-27-treated immature DCs can affect expression of T_reg_-associated molecules on CD4^+^ T cells *in vivo*. To test whether or not IL-27-treated immature DCs can modulate expression of T_reg_-associated molecules on CD4^+^ T cells *in vivo*, MOG peptide-pulsed immature DCs incubated with IL-27 or without IL-27 treatment were i.v. transferred into EAE mice shown in Figure 4 at day 11, 14, and 17 after immunization. Lymphocytes were isolated from mice which are i.v transferred with IL-27-treated DCs or immature DCs without IL-27 treatment shown in Figure 4 at day 25. Expression of T_reg_-associated molecules on CD4^+^ T cells was detected using flow cytometry. Our results demonstrated that there is no difference in expression of T_reg_ -associated molecules, including CD25, CD127, FoxP3, GITR, and 3G11, on CD4^+^ T cells isolated from mice that are i.v. transferred with IL-27-treated immature DCs or DCs without IL-27 incubation (Figures 5A–E). Our data suggest that IL-27-treated immature DCs do not affect development of T_regs_ *in vivo*.

### IL-27-treated mature DCs do not affect expression of T_reg_-associated molecules on CD4^+^ T cells *ex vivo*

**Figure 6:**
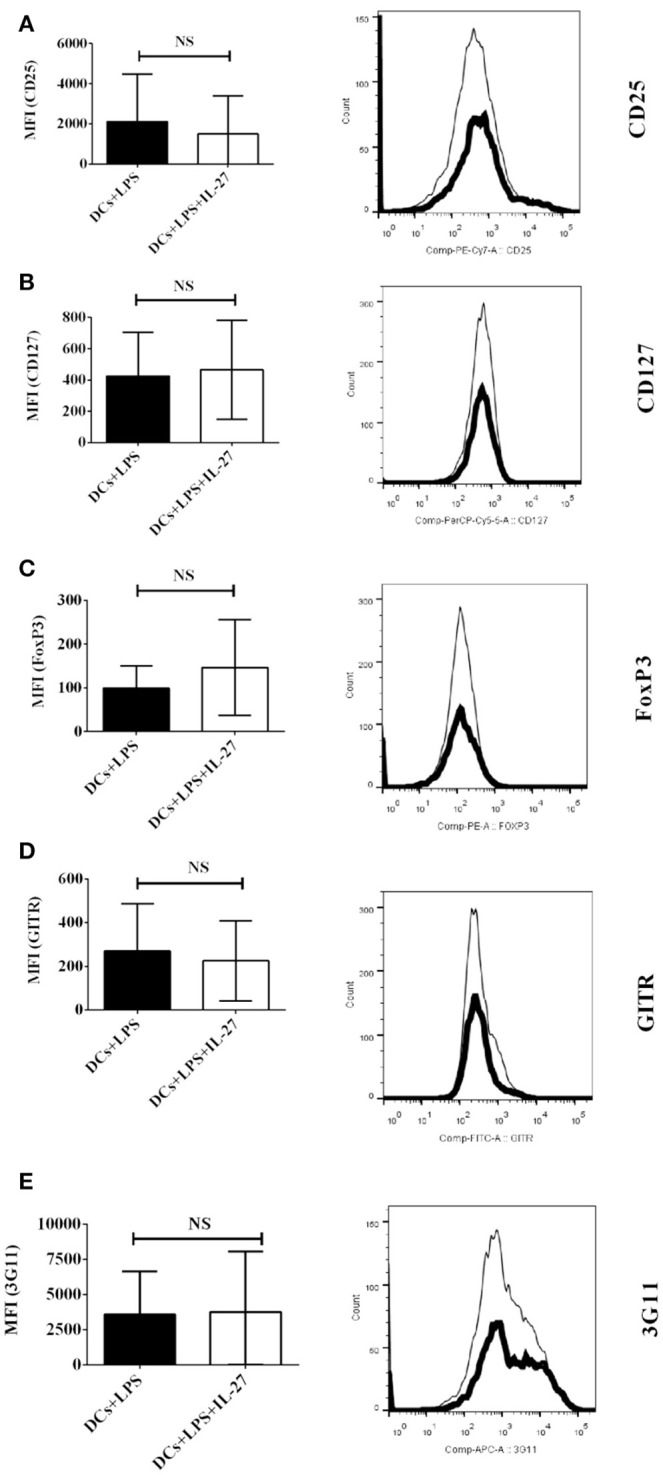
IL-27-treated mature DCs induced by LPS do not affect expression of T_reg_-associated molecules on CD4^+^ T cells *ex vivo*. LPS-stimulated DCs (Thin line) or IL-27-treated mature DCs induced by LPS (Thick line) were i.v. transferred into mice shown in Figure 4. Spleen cells were isolated and re-stimulated with MOG peptide (0.1 μM) and IL-2 (1 ng/ml) for 72 hrs. The expression of CD25 **(A)**, CD127 **(B)**, FoxP3 **(C)**, GITR **(D)**, and 3G11 **(E)** on CD4^+^ T cells is shown. Error bars indicated in this figure represent mean and SD of MFI of T_reg_-associated molecules expressing on CD4^+^ T cells in three independent experiments (*n* = 3, *t* test, P_A_ = 0.7015; P_B_ = 0.8555; P_C_ = 0.4659; P_D_ = 0.7642; P_E_ = 0.9505; NS, no significant difference).

We have testified that IL-27-treated mature DCs do not affect expression of T_reg_-associated molecules on CD4^+^ T cells *in vitro* (Figure 2), however, it is still unclear that whether or not IL-27-treated mature DCs can modulate expression of T_reg_-associated molecules on CD4^+^ T cells *in vivo*. To determine whether IL-27-treated mature DCs can modulate expression of T_reg_-associated molecules on CD4^+^ T cells *in vivo*, IL-27-treated mature DCs induced by LPS or mature DCs without IL-27 treatment were i.v. transferred into EAE mice shown in Figure 4 at day 11, 14 and 17 after immunization. Protein expression of CD25, CD127, FoxP3, GITR, and 3G11 on CD4^+^ T cells was tested using flow cytometry. The experimental data show that i.v. transfer of IL-27-treated mature DCs or mature DCs without IL-27 treatment failed to modulate expression of T_reg_-associated molecules on CD4^+^ T cells (Figures 6A–E). Our results suggest that IL-27-treated mature DCs do not affect development of T_regs_ through regulating expression of T_reg_-associated molecules on CD4^+^ T cells *in vivo*.

### IL-27-treated mature DCs facilitate development of CD4^+^ CD127^+^3G11^+^ T_reg_ subset *ex vivo*

**Figure 7:**
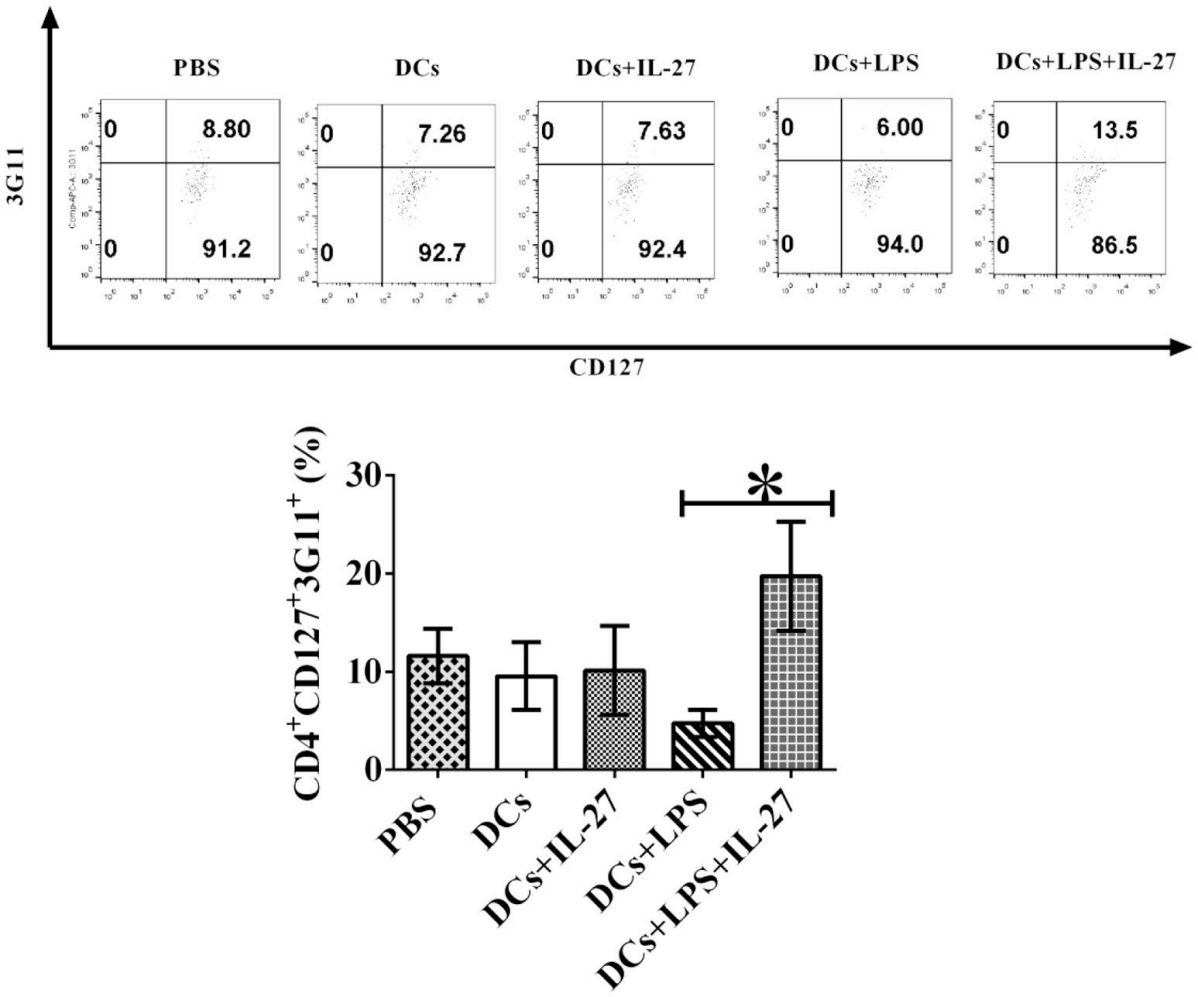
IL-27 facilitates LPS-stimulated mature DC-mediated development of CD4^+^CD127^+^3G11^+^ T_reg_ subset *ex vivo*. Bone marrow-derived DCs were pulsed with MOG peptide and treated with IL-27 (20 ng/ml, 72 h) (DCs+IL-27) or LPS (DCs+LPS) (1 μg/ml, 24 h) or both LPS and IL-27 (DCs+LPS+IL-27). These DCs were then i.v. transferred into mice with EAE shown in Figure 4. Mice treated with PBS are control. Spleen cells were isolated and re-stimulated with MOG peptide (0.1 μM) and IL-2 (1 ng/ml) for 72 h. Cells were collected and CD4^+^CD25^+^FoxP3^+^GITR^+^ T_regs_ were gated. The frequency of CD127^+^3G11^+^ cells is demonstrated. T lymphocytes incubated with isotype control antibodies are isotype control. Error bars indicated in this figure represent mean and SD of frequency of CD4^+^CD127^+^3G11^+^ cells in three independent experiments [*n* = 3, *t* test, P_(DC, DC+IL−27)_ = 0.8682; P_(DC+LPS, DC+LPS+IL−27)_ = 0.0105].

Our results of *in vitro* assay demonstrated that IL-27-treated mature DCs elicit development of CD4^+^ CD127^+^3G11^+^ T_reg_ subset (Figure 3), however, it is unknown whether or not IL-27-treated mature DCs also can facilitate development of CD4^+^ CD127^+^3G11^+^ T_reg_ subset *in vivo*. To detect whether or not IL-27-treated mature or immature DCs can regulate development of T_reg_ sub-populations *in vivo*, LPS-induced mature DCs and immature DCs without LPS stimulation were pulsed with MOG peptide and incubated with or without IL-27 treatment. IL-27-treated immature/mature DCs or immature/mature DCs without incubation with IL-27 were i.v. transferred into EAE mice shown in Figure 4 at day 11, 14 and 17 after immunization. Lymphocytes were isolated from mice that had been i.v. transferred with IL-27-treated immature/mature DCs or immature/mature DCs without IL-27 treatment shown in Figure 4 at day 25. Our results indicate that immature DCs incubated with or without IL-27 treatment did not modulate development of CD4^+^3G11^+^CD127^+^ T_reg_ subset but that mature DCs treated with IL-27 facilitated development of CD4^+^ CD127^+^3G11^+^ T_reg_ sub-populations *ex vivo* (Figure 7). Our data suggest that IL-27-treated mature DCs may block autoimmunity (Figure 4) through upregulation of CD4^+^CD127^+^3G11^+^ T_regs_ development *in vivo*.

## Discussion

IL-27 is a novel cytokine whose immune function has not yet been fully elucidated. Chiyo et al. reported that tumor cells expressing IL-27 activate CD4^+^ T helper cells, CD8^+^ cytotoxic T lymphocytes and natural killer cells (21, 22). IL-27 shows an effect of anti-tumor immunity as a possible therapeutic target for cancer (21). IL-27 plays an important role in T cell differentiation and regulation of T cell-mediated immune responses. For example, IL-27 produced by dendritic cells facilitates the polarization of T helper 1 cells in Lewis rats (23). Harker et al. recently found that IL-27-mediated signaling is necessary for anti-viral immunity (24). Moreover, IL-27 promotes differentiation of T helper 17 cells *in vivo* (25). Our data also indicate that IL-27 inhibits LPS-induced mature DC-mediated immune tolerance. These data suggest that IL-27 is a pro-inflammatory cytokine and a positive regulator in T cell-mediated immune responses.

Interestingly, the experimental data also indicate that IL-27 is an anti-inflammatory cytokine and inhibits development of autoimmunity *in vivo*. For instance, Mascanfroni et al. reported that IL-27 induces expression of CD39 on DCs and blocks development of T helper 1 and 17 cells to inhibit EAE induction (26). Tsoumarkidou et al. also found that tolerogenic CD1c^+^ DCs regulate development of T_regs_ via IL-27/IL-10 inducible co-stimulatory ligands (27). Rostami et al. have published data showing that the induction of peripheral tolerance is dependent on IL-27-mediated signal transduction pathway in DCs (28). These data suggest that regulatory mechanisms of IL-27 in the immune system are extremely complex and that IL-27 may play a dual role in equilibrium between autoimmunity and immune tolerance. Our results demonstrate that IL-27 does not affect immature DC-mediated immune responses but that it facilitates mature DC-mediated CD4^+^CD127^+^3G11^+^ T_reg_ development. This suggests that the immune function of IL-27 on DCs is dependent on their maturation.

The cellular and molecular regulatory mechanisms of IL-27 have been recently investigated. For example, it is known that IL-27 can modulate development and biological function of T helper 17 cells, dendritic cells, NK cells and neutrophils (22, 25, 29, 30). IL-27 produced by DCs is necessary for trafficking of T_regs_ to locate in tumor (31), and pulmonary CD1c^+^ DC-mediated development of T_regs_ is dependent on IL-27/IL-10/inducible costimulator ligand (27). IL-27 also facilitates development of T_reg_ and induces immune tolerance *in vivo* (32). By contrast, our data indicate that IL-27-treated mature DCs elicit development of CD4^+^CD127^+^3G11^+^ T_reg_ subset and inhibits mature DC-mediated immune tolerance *in vivo* (Figure 7).

The immunological significance of this study is that we find a new subset of CD4^+^ T_regs_ mediated by 3G11 and CD127. Biological function of CD4^+^CD127^+^3G11^+^ T_regs_ may be different from that of conventional CD4^+^ T_regs_. Previous studies showed that CD4^+^ T_regs_ is a negative regulator of autoimmunity. By contrast, the frequency of CD4^+^CD127^+^3G11^+^ T_regs_ increases in mice in which LPS-stimulated DC-mediated immune tolerance is inhibited. This new sub-population of T_regs_ may be a positive regulator which facilitates T cell-mediated immune responses *in vivo*. This has never been reported.

IL-27 can act as both pro-and anti-inflammatory cytokine, however, molecular mechanisms of IL-27 to modulate autoimmunity and immune tolerance have not yet been fully elucidated. Our data indicated that IL-27 does not affect immature DC-mediated immune responses, however, IL-27 can block immune tolerance induced by LPS-stimulated mature DCs. IL-27 acts as a pro-inflammatory cytokine to inhibit immune function of LPS-treated mature DCs. Interestingly, IL-27 only elicits development of CD4^+^CD127^+^3G11^+^ T_regs_ mediated by mature DCs induced by LPS. IL-27 does not affect that of T_reg_ subset mediated by immature DCs. It may be dependent on maturation of DCs whether IL-27 plays a role of pro- or anti-inflammatory cytokine *in vivo*.

The interesting question is how IL-27 regulates LPS-stimulated mature DC-mediated immune tolerance *in vivo*. There is little amount of data to reveal it. The molecular mechanisms of IL-27 to block immune tolerance mediated by LPS-treated mature DCs should be investigated in the future so that a new immune therapy using CD4^+^CD127^+^3G11^+^ T_reg_ sub-population can be designed to treat human diseases.

In summary, Our results imply that CD4^+^CD127^+^3G11^+^ cells may be a type of positive T_regs_ which are different from conventional CD4^+^ T_regs_ which inhibit autoimmunity *in vivo*. This new subset of T_regs_ are CD127 positive cells and conventional CD4+ T_regs_ express CD127 with low level, although both of them are CD4^+^CD25^+^FoxP3^+^GITR^+^ cells. Biological function of CD4^+^CD127^+^3G11^+^ T_regs_ may be different from that of conventional CD4^+^CD25^+^CD127^low^FoxP3^+^GITR^+^ T_regs_. Immune functions of this new CD4^+^CD127^+^3G11^+^ T_reg_ subset should be investigated in future studies.

## Ethics statement

All experimental procedures were approved by the Institutional Animal Care and Use Committee of Thomas Jefferson University.

## Author contributions

FZ designed and conducted experiments for this research project. G-XZ and AR supervised the research and reviewed the manuscript.

### Conflict of interest statement

The authors declare that the research was conducted in the absence of any commercial or financial relationships that could be construed as a potential conflict of interest.

## Abbreviations

APC: Allophycocyanin
CD: Cluster of differentiation
CFA: Complete Freund's adjuvant
DC: Dendritic cell
EAE: Experimental autoimmune encephalomyelitis
FCS: Fetal Calf Serum
Fig: Figure
GM –CSF: Granulocyte-macrophage colony-stimulating factor
IL: Interleukin
i. p.: Intraperitoneal
i.v.: Intravenous
KLH: Keyhole limpet hemocyanin
LPS: Lipopolysaccharide
MOG: Myelin oligodendrocyte glycoprotein
2-ME: 2-mercaptoethanol
MS: Multiple sclerosis
PBS: Phosphate-buffered saline
pDCs: Plasmacytoid dendritic cells
PI: Propidium Iodide
PT: Pertussis toxin
s.c.: Subcutaneous
SD: Standard deviation
SEM: Standard error of arithmetic mean
Th: T helper cells.
